# Stage‐dependent involvement of ADAM10 and its significance in epileptic seizures

**DOI:** 10.1111/jcmm.14307

**Published:** 2019-05-13

**Authors:** Xu Zhou, Hua Tao, Yujie Cai, Lili Cui, Bin Zhao, Keshen Li

**Affiliations:** ^1^ Clinical Research Center Affiliated Hospital of Guangdong Medical University Zhanjiang China; ^2^ Department of Neurology Affiliated Hospital of Guangdong Medical University Zhanjiang China; ^3^ Guangdong Key Laboratory of Age‐related Cardiac and Cerebral Diseases Affiliated Hospital of Guangdong Medical University Zhanjiang China; ^4^ Institute of Neurology Affiliated Hospital of Guangdong Medical University Zhanjiang China; ^5^ Stroke Center, Neurology & Neurosurgery Division, Clinical Medicine Research Institute & the First Affiliated Hospital Jinan University Guangzhou China

**Keywords:** ADAM10, Alzheimer's disease, amyloidogenic processes, cortical dysplasia, epilepsy

## Abstract

The prevalence of epileptic seizures in Alzheimer's disease (AD) has attracted an increasing amount of attention in recent years, and many cohort studies have found several risk factors associated with the genesis of seizures in AD. Among these factors, young age and severe dementia are seemingly contradictory and independent risk factors, indicating that the pathogenesis of epileptic seizures is, to a certain extent, stage‐dependent. A disintegrin and metalloproteinase domain‐containing protein 10 (ADAM10) is a crucial α‐secretase responsible for ectodomain shedding of its substrates; thus, the function of this protein depends on the biological effects of its substrates. Intriguingly, transgenic models have demonstrated ADAM10 to be associated with epilepsy. Based on the biological effects of its substrates, the potential pathogenic roles of ADAM10 in epileptic seizures can be classified into amyloidogenic processes in the ageing stage and cortical dysplasia in the developmental stage. Therefore, ADAM10 is reviewed here as a stage‐dependent modulator in the pathogenesis of epilepsy. Current data regarding ADAM10 in epileptic seizures were collected and reviewed for potential pathogenic roles (ie amyloidogenic processes and cortical dysplasia) and regulatory mechanisms (ie transcriptional and posttranscriptional regulation). These findings are then discussed in terms of the significance of the stage‐dependent functions of ADAM10 in epilepsy. Several potential targets for seizure control, such as candidate transcription factors and microRNAs that regulate ADAM10, as well as potential genetic screening tools for the early recognition of cortical dysplasia, have been suggested but must be studied in more detail.

## 1 INTRODUCTION

Alzheimer's disease (AD) is a major neurocognitive disorder that has attracted an increasing level of concern with the increasing age of the global population. Interestingly, epileptic seizures are prevalent in AD patients,^1^, ^2^ but the underlying mechanisms of this phenomenon are unclear. As AD‐related epilepsy is an important form of late‐onset epilepsy in clinical practice, the elucidation of its pathogenesis could contribute to the existing understanding of epileptic seizures.

As a crucial molecule in AD pathology, a disintegrin and metalloproteinase domain‐containing protein 10 (ADAM10) inhibits the formation of amyloid β (Aβ) via the competitive cleavage of amyloid precursor protein (APP) into nontoxic products, thus displaying a protective effect against AD.^3^ Intriguingly, transgenic mice with dominant‐negative Adam10 (Adam10dn) display low thresholds for epileptic seizures as well as cognitive impairments in AD models.^4^ Moreover, amyloidogenic processes inhibited by ADAM10, including Aβ aggregation, have been confirmed to induce epileptic seizures,^5^, ^6^, ^7^ supporting a role for ADAM10 in late‐onset epilepsy. In addition, ADAM10 functions in brain development, and the loss of function of this molecule can result in cortical dysplasia, followed by refractory seizures,^8^, ^9^, ^10^ indicating that ADAM10 is also involved in late‐onset epilepsy. Hence, ADAM10 most likely functions as a stage‐dependent modulator in the pathology of epilepsy.

We have observed that severe cognitive decline and young age are independent risk factors for epileptic seizures in AD patients,^11^, ^12^, ^13^, ^14^, ^15^ yet, the former usually appears in the elderly because of chronic Aβ aggregation, which is against the predisposition to epileptic seizures at younger ages. Interestingly, this seeming contradiction conforms to the stage‐dependent involvement of ADAM10 in amyloidogenic processes and cortical dysplasia. Hence, this review discusses the current evidence for the role of ADAM10 in epileptic seizures. We subsequently review the potential pathogenic and regulatory mechanisms of ADAM10 and then discuss the stage‐dependent significance of ADAM10 in epilepsy.

## 2 CURRENT EVIDENCE FOR ADAM10 IN EPILEPSY

### 2.1 Clinical clues from the concurrence of AD and epilepsy

Traditionally, AD and epilepsy do not belong to the same classification of brain disorders, which are individually characterized by cognitive decline and recurrent seizures respectively. However, an increasing amount of evidence has supported their intriguing relationship in the past decades. In fact, seizure morbidity ranges from 1%‐22% of AD patients, and seizure incidence varies from 4.8 to 11.9/1,000 person‐years in AD patients, which is 2‐ to 6‐fold higher than the rates in age‐matched normal individuals.^1^ In a large cohort of patients with autosomal dominant early‐onset AD, the seizure incidence reached nearly 50% after an average follow‐up of 8.4 years.^2^ Seizures and reduced seizure thresholds have been further confirmed in transgenic mouse models for familial AD.^16^ In addition, Down syndrome (DS) is a disease that simultaneously possesses the typical symptoms of AD and epilepsy, with epileptic seizures usually emerging over the course of dementia.^15^ All of these findings suggest the prevalence of epileptic seizures in AD.

On the other hand, community‐dwelling elders with partial epilepsy have been found to exhibit cognitive decline in comparison to healthy controls and an even greater decline in executive function than do patients with mild cognitive impairment, which is known as a precursor to AD.^17^, ^18^ As a pathological marker of AD, Aβ aggregation in resected cerebral cortices of patients with temporal lobe epilepsy was significantly greater than that in autopsy controls.^19^ In a 50‐year follow‐up of childhood‐onset epilepsy patients and matched controls, a high level of Aβ burden was observed in the prefrontal cortex, parietal cortex and posterior cingulate/precuneus of patients but not of controls,^20^ an observation that was similar to the findings in patients with preclinical AD.^21^, ^22^ In addition, antiepileptic treatments have been found to display a positive effect on cognitive function in animal and human studies.^23^ Hence, epileptic activities might be a driving factor for the incidence of AD in patients with epilepsy.

### 2.2 Emerging concern for stage‐dependent ADAM10 in epilepsy

Considering the concurrence of AD and epilepsy, concern has emerged in recent years regarding whether these conditions share certain pathogeneses, and clarifying this issue might contribute to understanding epileptic seizures from a novel perspective. Interestingly, a series of cohort studies found risk factors associated with the genesis of seizures in AD, particularly the seemingly contradictory and independent risk factors of young age and severe dementia.^11^, ^12^, ^13^, ^14^, ^15^

Indeed, patients with genetic/familial forms of AD who carry mutations in APP, presenilin‐1 (PS1) and −2 (PS2) are particularly prone to epileptic seizures. Aβ, acting as a downstream molecule correlating with the severity of dementia, likely plays a key role in epilepsy. Moreover, the percentage of patients with epileptic seizures has been found to reach one‐third in patients with APP duplications, but no patient was found to have APP mutations over a 5‐year observation period.^2^ Intriguingly, little neuronal loss appears in APP transgenic mice with spontaneous seizures, implying a prominent role of Aβ in synchronized abnormal discharges by a direct excitatory effect on brain networks rather than end‐stage degeneration.^1^, ^24^ Despite these findings, the severity of dementia often increases with age because of chronic Aβ aggregation, which is not inconsistent with the predisposition to epileptic seizures at younger ages, indicating the involvement of other pathogenic effects in early‐onset epilepsy. Among various hypotheses, ADAM10 is a promising candidate to bridge the apparent contradiction in risk factors for seizures in AD because of its stage‐dependent involvement in both amyloidogenic processes and cortical dysplasia. Further investigation of this hypothesis would assist in addressing whether certain pathogenic mechanisms are shared between AD and epilepsy.

### 2.3 Epileptic phenotypes of ADAM10 in transgenic models

ADAM10 is a key α‐secretase responsible for physiological shedding of the APP ectodomain, for which it competes and thereby inhibits Aβ deposition owing to β‐site cleavage. Thus, ADAM10 plays a key role in AD pathology. Notably, transgenic mice with Adam10dn/APP experience more severe seizures and longer recovery times. These animals also show more significant degeneration in the hippocampal region compared to transgenic mice that moderately overexpress Adam10 (Adam10 mo)/APP and APP mice with endogenous ADAM10 levels.^4^ Furthermore, the loss of function of ADAM10 was observed to be related to increased seizure‐associated mortality after weaning in an ADAM10 conditional knockout (cKO) mouse model.^25^ In addition, increased β‐site cleavage products and epileptic seizures were observed in APPSWE mice that were developed to simulate extracellular Aβ aggregation.^26^ All of these findings support the notion that ADAM10 could protect against epileptic seizures in AD.

## 3 POTENTIAL PATHOGENIC ROLES OF ADAM10 IN EPILEPSY

### 3.1 Amyloidogenic processes

Because of the competitive role of ADAM10 in inhibiting Aβ aggregation, the decreased function of this molecule seems to be a key factor for amyloidogenic processes.^27^, ^28^, ^29^, ^30^, ^31^ Thus, interest has increased into whether the Aβ burden mediated by ADAM10 is involved in epileptic seizures as well as in cognitive decline in AD. In fact, a correlation between the frequency of epileptiform‐like discharges and the number of Aβ plaques has been observed in an APP/PS1 mouse model of AD. Moreover, epileptic activity appeared when *N*‐(2‐chloroethyl)‐*N*‐ethyl‐bromobenzylamine was employed to form Aβ plaques in the cortex.^5^ Hypersynchronous activities underlying epileptic seizures even preceded Aβ plaques and memory impairment and could be improved by passive immunization with an anti‐human APP/Aβ antibody,^6^, ^7^ indicating that early stages of aggregation before the development of Aβ plaques, such as the formation of Aβ tetramers, might initiate the process of epilepsy pathology.

Excessive excitatory transmission is necessary in the process of epileptic seizures, and Aβ deposition is significantly increased in the temporal cortex and the hippocampus of patients with refractory epilepsy.^32^ Notably, γ‐aminobutyric acid (GABA) terminals are in contact with the somas and proximal axons of the cerebral cortex in layers II‐VI but are replaced by Aβ deposits around the membranes of neurons, primarily pyramidal cells, in AD.^33^ As these GABA synapses exert inhibitory effects on the action potentials of excitatory pyramidal cells, the loss of function of these synapses would lead to the hyperactivity of the neurons surrounded by Aβ deposits. These previous studies have supplied a clue regarding the pathological role of Aβ‐mediated excitotoxicity in epileptic seizures.

Recently, a humanized monoclonal antibody, solanezumab, which was designed to increase Aβ clearance, failed to improve the cognitive decline in a double‐blind, placebo‐controlled, phase 3 trial with a total of 2129 AD patients.^34^ Considering that epileptic seizures are comorbid with dementia in AD, we must doubt the role of Aβ burden in epilepsy. Interestingly, transgenic mice overexpressing the APP intracellular domain (AICD) and the binding partner of this domain, Fe65, displayed abnormal spiking episodes. Moreover, these mice were susceptible to a seizure‐causing stimulus. Importantly, this phenomenon was not observed in transgenic mice overexpressing Aβ and harbouring a mutation (D664A) in the AICD. These results indicated that in addition to Aβ in amyloidogenic processes, AICD might be another modulator associated with epileptic seizures. Hence, targeting the integral modulation of Aβ and the AICD could be a strategy for treating epilepsy, and the upstream molecule ADAM10 could be a promising candidate for modulating amyloidogenic processes.

### 3.2 Cortical dysplasia

In addition to APP, many other ADAM10 substrates have been reported,^35^ and many more could remain undiscovered given the extensive effects ADAM10 exerts via the shedding of the ectodomain.^36^ These known and potential substrates speak to the variety of roles of ADAM10 in biological activities. In light of the complex interaction between ADAM10 substrates in vivo, it is necessary to comprehensively describe the major biological effects of ADAM10, as well as its classical role in amyloidogenic processes.

Using brain samples from transgenic Adam10dn mice, microarray analyses were performed to evaluate the influence of insufficient ADAM10 on mRNA expression profiles in the brain. A total of 143 genes were observed to be differentially expressed in transgenic mice compared with the wild‐type mice, with most genes being implicated in nervous system development, neuronal projections and synaptic transmission.^37^ The crucial role of ADAM10 in brain development has been repeatedly described in recent years, and cortical dysplasia is one of the most common aetiologies of intractable epilepsy, especially in early‐onset patients.^38^, ^39^, ^40^, ^41^ These previous results imply the involvement of ADAM10 in epilepsy via the modulation of cortical development.

Compared with amyloidogenic processes in elderly individuals, ADAM10‐mediated cortical dysplasia is distinct and is most likely caused by a non‐amyloidogenic pathogenic mechanism in early‐onset epilepsy. Thus, we reviewed the major progress that has been made in understanding the role of ADAM10 in this condition.

#### 3.2.1 Radial migration and lamination

Radial migration of cortical neurons from neurogenic regions to superficial layers is a key process in the morphogenesis of the cerebral cortex.^42^ The Notch intracellular domain (NICD) is essential for radial migration of cortical neurons,^43^ and ADAM10 is a key enzyme involved in the second cleavage of the Notch extracellular juxtamembrane region.^44^ Magnetic resonance imaging revealed that ADAM10 expression is related to the thickness of gray matter in the human brain.^8^ Moreover, radial migration was shown to be impaired in ADAM10‐deficient neurons, and only the NICD, but not the intracellular domains of other substrates, such as APP and *N*‐cadherin, could significantly restore this impairment.^9^ These data indicate a key role of ADAM10 in the radial migration of cortical neurons via cleavage of the NICD.

As microtubules pull neuronal somas, they are the basic structures necessary for radial migration.^45^ Previous evidence demonstrated that newborn cortical neuron motility is significantly impaired in the absence of ADAM10 and that disrupted microtubules are related to the decreased expression of acetylated tubulin and microtubule‐associated proteins.^9^ Intriguingly, the NICD/RBPJ complex can enhance the promoter activity of doublecortin, a modulator of microtubule cytoskeletons, and up‐regulate the expression of doublecortin in neurons. Similar to the NICD, the overexpression of doublecortin was shown to significantly rescue the cortical neuron motility and reversed migratory defects caused by ADAM10 KO.^9^ Hence, doublecortin is most likely a downstream molecule in the ADAM10/Notch pathway's involvement in the radial migration of cortical neurons.

In addition to radial migration, cortical lamination is also necessary for cortical development and depends on the normal differentiation of neural stem/progenitor cells. To circumvent the early embryonic lethality of Adam10 KO mice because of multiple developmental defects, such as disturbed somitogenesis and severe vascular defects,^46^ the Adam10 cKO technique was used to investigate the function of this protein in the brain by limiting its inactivation to neural progenitor cells (NPCs) and NPC‐derived neurons and glial cells. cKO mice die perinatally with a disrupted neocortex and severely reduced ganglionic eminence owing to precocious differentiation, resulting in the early depletion of progenitor cells. Furthermore, neurospheres derived from the cKO mice have disrupted organization and more neurons at the expense of astrocytes. These findings supported the role of ADAM10 in cortical lamination via the regulation neural stem/progenitor cell differentiation.

As Notch activity is important for cell fate decisions during neurogenesis,^47^ it would be interesting to know whether this pathway is involved in the premature neuronal differentiation of Adam10 cKO mice. Indeed, an accumulation of Notch‐1 cleavage product via furin was evident in the absence of ADAM10, but a strong reduction in the generation of the NICD was observed in Adam10 cKO mice. Further investigation showed reductions in Hes1, Hes5, Hey1 and Hey2. These factors are basic helix‐loop‐helix transcription factors (TFs) that repress proneural genes, such as Ascl1 and neurogenin, themselves major downstream molecules of Notch signalling.^48^ This evidence supports the notion that disorganized laminar architecture in the absence of ADAM10 could be mediated by the Notch‐1 pathway.

#### 3.2.2 Neurite and synapse formation

Neurite outgrowth is a fundamental process for formation of cortical neuron circuits in the brain. Current evidence suggests that IgLON family proteins can be released into the extracellular space via metalloproteinase activity, thereby affecting neuronal maturation.^49^ In particular, neurons lacking Negr1, a member of the IgLON family, show reductions in both dendrite length and number, indicating that Negr1 promotes neuronal tree growth.^50^ Intriguingly, ADAM10 can release Negr1 from neuronal membranes, and the pharmacological inhibition of ADAM10 was demonstrated to lead to defects in neurite maturation. Importantly, this effect could be rescued by soluble Negr1 administration.^10^ Hence, ADAM10 can regulate neurite outgrowth via the ectodomain shedding of Negr1.

Neuronal cell adhesion molecule (NCAM) is an important regulator of neuronal outgrowth and synaptic plasticity. EphrinA/EphA‐dependent axon repulsion is crucial for synapse formation, and ephrinA5/EphA3 can trigger NCAM proteolysis via ADAM10 to promote growth cone collapse.^51^ In cortical neuron cultures, the soluble extracellular domain of NCAM (NCAMed) was demonstrated to inhibit NCAM‐dependent neurite branching and outgrowth.^52^ However, transgenic mice overexpressing soluble NCAMed displayed reduced GABAergic synapses and altered behavioural phenotypes.^52^ These results indicate that excess levels of soluble NCAMed because of ADAM10 activity can reduce the perisomatic innervation of cortical neurons by inhibiting synapse formation during cortical development.

Synapses are the basic units of information transmission, processing and integration in the nervous system. *N*‐cadherin plays important roles during dendritic arborization, axonal guidance and synaptogenesis. In particular, *N*‐cadherin at synaptic sites is involved in the regulation of cell‐cell adhesion and plasticity control. Recent studies have indicated that *N*‐cadherin can be shed by ADAM10, and impairing the localization and activity of ADAM10 at synaptic sites could result in a decreased ability to process *N*‐cadherin, leading to the accumulation of full‐length *N*‐cadherin, increases in spine head width, and abnormal modifications of the number and function of glutamatergic AMPA receptors.^53^ In addition, the roles of PLD1 and PACAP38 in synaptogenesis were also affected by ADAM10‐mediated *N*‐cadherin cleavage.^54^

Neurexins are a type of synaptic cell adhesion molecule involved in the development and maturation of neuronal synapses. Notably, the neurexin Nrxn3β can be processed by ADAM10, and the shedding of the ectodomain of Nrxn3β releases a soluble fragment that increases the spine density of newborn hippocampal neurons,^55^ indicating that ADAM10 modulates synaptogenesis via ectodomain shedding of Nrxn3β, as well as Negr1, NCAM and *N*‐cadherin.

**Figure 1:**
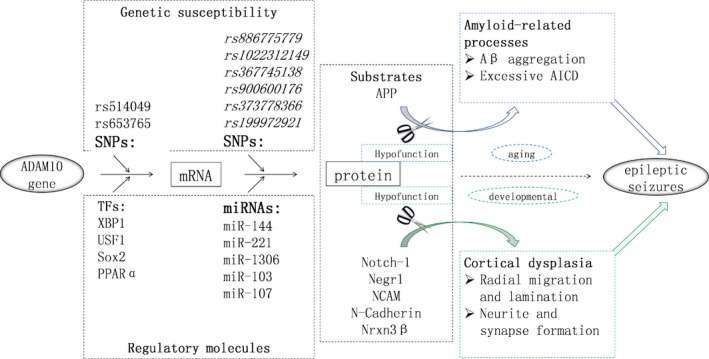
Stage‐dependent involvement of ADAM10 and its modulation in epileptic seizures. On the basis of previous research and functional analysis of gene structure, SNPs in the predicted ADAM10 promoter and 5′‐untranslated region (UTR) are potential sites at which ADAM10 levels can be controlled transcriptionally and posttranscriptionally respectively. Such SNPs could therefore be the basis of ADAM10‐related genetic susceptibility to epilepsy. In accordance with current evidence, the shown TFs and miRNAs likely regulate ADAM10 at transcriptional and posttranscriptional levels respectively. Decreased ADAM10 function in the ageing stage could result in abnormal ectodomain shedding of APP, which is closely associated with amyloidogenic processes and related epileptic seizures, which tend to present in patients with late‐onset AD. Meanwhile, decreased ADAM10 function in the developmental stage could result in insufficient ectodomain shedding of its substrates, such as Notch‐1, Negr1, NCAM, *N*‐Cadherin and Nrxn3β. These effects are usually associated with cortical dysplasia and related epileptic seizures that tend to occur in early‐onset cases

ADAM10 plays a key role in ectodomain shedding. Thus, the functions of ADAM10 depend on the biological effects of its substrates. Several substrates have been associated with epileptic seizures via their effects on amyloidogenesis in the ageing stage and cortical dysplasia in the developmental stage (Figure 1). In addition, more substrate candidates have been found through mRNA and protein expression profiling of ADAM10‐deficient neurons and brain tissues.^36^, ^37^ These data together indicate that the pathogenic mechanisms of ADAM10 in epilepsy are likely complex and must be further explored.

## 4 REGULATORY MECHANISMS OF ADAM10 EXPRESSION

Although ADAM10 has a protective effect against epileptic seizures in AD transgenic models, increasing expression above its endogenous level is not associated with increased protection against epilepsy. In contrast, transgenic mice with high levels of ADAM10 exhibit more seizures and stronger neuronal damage and inflammation than do wild‐type mice and those with moderate levels of ADAM10.^4^ These findings indicate that excessive ADAM10‐mediated cleavage might counterbalance the antiepileptic effects of ADAM10. Therefore, to avoid the potentially seizure‐inducing effects of ADAM10 cleavage products, the precise regulation of ADAM10 expression is necessary. The transcriptional and posttranscriptional regulatory mechanisms of ADAM10 are reviewed below.

### 4.1 Transcriptional level

In the human genome, ADAM10 is located at 15q21.3 and has a length of 154 744 nucleotides (nts). Within 2 200 nts upstream of the translational start site, a classical CAAT‐box at −480 nt in the 5′‐flanking region of the human ADAM10 gene was found via computational sequence analysis, representing a potential regulatory element required to initiate gene transcription.^56^ A series of deletion mutations further identified −508 nt to −300 nt as the core promoter region. Interestingly, a potential Maz binding site (−491 nt), a CAAT‐box (−479 nt), a Sp1 binding site (−366 nt) and an USF element (−317 nt) were observed in this region. Removing these binding sites strongly decreased the promoter activity of ADAM10,^56^ indicating that this region is required for ADAM10 transcription.

In the human genome, numerous genetic variations exist around the core promoter region of ADAM10, but most of these are rare mutations. Only rs514049 A > C and rs653765 G > A (−630 nt and −279 nt upstream of the translation initiation site of ADAM10 respectively) are common variations that may potentially confer genetic susceptibility to ADAM10‐related diseases (Figure 1). Intriguingly, linkage disequilibrium between these two sites was observed via haplotype analysis, and compared to the A‐G haplotype (rs514049‐rs653765), the C‐A haplotype showed decreased levels of soluble APPα in AD but higher levels than those in healthy controls.^57^ This finding implies the differential involvement of TFs in ADAM10 transcriptional regulation. Remarkably, the C‐A haplotype (rs514049‐rs653765) also showed an association with epilepsy.^58^ Thus, future efforts should be made to clarify the involvement of TFs in the haplotype.

Considering that BACE1 competes with ADAM10 to shift APP processing from protective α‐ to neurotoxic β‐cleavage, a library composed of 704 expression plasmids encoding human TFs was employed and ultimately found that XBP1 is an exclusive candidate for ADAM10 regulation compared with BACE1. In addition, numerous TFs or inducers, such as XBP1,^59^ USF1,^60^ Sox2,^61^ PPARα,^62^ melatonin^63^ and SIRT1, 64 have been reported to promote ADAM10 transcription and to protect against amyloidogenic processes (Figure 1). However, whether these TFs also function in non‐amyloidogenic processes, particularly cortical dysplasia, remains unclear. Among humans, mice and rats, the sequence identity is as high as 78% within 500 nts upstream of ADAM10 translational start sites. In particular, the regulatory elements are highly evolutionarily conserved between Homo sapiens and *Rattus norvegicus*, which would facilitate cross‐species experiments involving transcriptional modulation of ADAM10.

### 4.2 Posttranscriptional level

#### 4.2.1 3′‐UTR regulation of ADAM10 expression

According to the integrated proteomics of normal tissues and cell lines from ProteomicsDB, MaxQB, and MOPED databases, ADAM10 is widely expressed in human blood as well as in the human immune, nervous, musculoskeletal and other internal systems. Consistent with this broad expression pattern, ADAM10 plays extensive roles in a variety of human diseases, such as brain disorders, immune system dysfunction and cancer.^65^ Hence, the non‐specific regulation of ADAM10 expression for seizure control most likely leads to off‐target effects on other biological activities.

MicroRNAs (miRNAs) are noncoding RNA molecules that suppress the translation of target mRNAs based on the complementary pairing principle between miRNA seed regions and the 3′‐UTR of their target mRNAs. A previous study showed increased promoter activity for an ADAM10 promoter‐luciferase construct compared to an ADAM10 promoter‐luciferase‐3′‐UTR construct in SH‐SY5Y cells, indicating the involvement of miRNAs in the posttranscriptional regulation of ADAM10.^57^ Several miRNAs targeting ADAM10 have been reported in pathological conditions, such as tumours and AD.^66^, ^67^, ^68^, ^69^, ^70^, ^71^, ^72^, ^73^, ^74^, ^75^, ^76^, ^77^, ^78^, ^79^, ^80^, ^81^, ^82^ However, the miRNAs involved in these pathological conditions nearly overlap; hence, the involvement of miRNAs in ADAM10 regulation is most likely disease specific, necessitating the development of disease‐specific strategies for treating ADAM10‐related diseases (Figure 1; Table 1).

**Table 1:** Validated miRNAs that target ADAM10 in human diseases

Validated miRNAs	Cells used in dual‐luciferase reporter gene assay	Relative expression levels in pathological conditions^a^	Biological effects by targeting ADAM10
Tumour‐related			
miR‐494	Tumour‐initiating cells of head and neck squamous cell carcinomas (HNSCC‐TICs)	Down‐regulated in local tumour tissues and metastatic lymph nodes in HNSCC	Inhibits HNSCC^73^
miR‐122	Hep3B cells	Down‐regulated in hepatocellular carcinoma (HCC) cells and tissues	Inhibits HCC^74^, ^75^
miR‐140‐5p	Tca8113 cells	No difference in tongue squamous cell carcinoma (TSCC) tissues	Inhibits TSCC^76^
miR‐448	HGC‐27 cells	Down‐regulated in gastric cancer (GC) tissues and cell lines	Inhibits GC^77^
miR‐449a	HepG2 cells	Down‐regulated in HCC tissues and cell lines	Inhibits HCC^78^
miR‐655‐3p	HCCLM3 and HepG2 cells	Down‐regulated in HCC tissues and cell lines	Inhibits HCC^79^
miR‐365	HepG2 cells	Down‐regulated in HCC tissues and cell lines	Inhibits HCC^80^
miR‐140‐5p	FaDu cells	Down‐regulated in hypopharyngeal squamous cell carcinoma (HSCC) tissues	Inhibits HSCC^81^
miR‐451	MDA‐MB‐231 cells	Down‐regulated in HNSCC tissues	Inhibits HNSCC^82^
miR‐320a	Gastric cells	Down‐regulated in gastric cancer	Inhibits cell growth and chemosensitivity in gastric cancer^83^
AD‐related			
miR‐144	SH‐SY5Y cells	Upregulated in elderly primate brains and AD patients	Mediates AD pathogenesis^79^
miR‐221	SH‐SY5Y cells	Downregulated in total blood obtained from AD patients	A new potential target for AD treatment^84^
miR‐1306			
miR‐103 miR‐107	SH‐SY5Y cells	Downregulated with age and in AD gray matter	Potential targets for AD treatment^85^
Others			
miR‐103a	HEK 293T cells	Upregulated in angiotensin II‐induced murine AAA specimens	Involvement in AAA progression^86^
miR‐155	HEK 293T cells	Upregulated in monocyte‐derived macrophages upon TLR3/4‐stimulation	Involvement in HIV‐1 inhibition^87^
miR‐144	N2A cells and HEK 293 cells	Upregulated in patients with traumatic brain injury (TBI) and experimental TBI rats	Mediates cognitive impairments following TBI^88^

Note

a the relative expression levels in pathological conditions were primarily compared to those in adjacent nontumour samples or matched normal samples.

miRNA/ADAM10 pathways have been primarily defined in tumours and AD but are undetermined in epilepsy. However, the crucial role of miRNAs in brain development has been demonstrated by the dramatic apoptotic phenotypes of mouse mutants with abolished miRNA synthesis.^83^ This result indicates that miRNAs are necessary for brain development and that abnormalities in miRNA expression might be involved in cortical dysplasia, which is usually followed by epileptic seizures. Thus, the role of miRNAs in ADAM10 regulation should be explored in the context of epilepsy pathology. According to miRNA target prediction tools, more than 200 miRNAs have the potential to target the human ADAM10 3′‐UTR 79 and are thus candidates for future experiments.

#### 4.2.2 5′‐UTR regulation of ADAM10 expression

According to the human genome, the 5′‐UTR of ADAM10 mRNA is composed of a 444‐nt sequence upstream of its translation initiation site. Importantly, the 5′‐UTR is often highly evolutionarily conserved and affects translation.^84^, ^85^ In a previous study, ADAM10 protein significantly increased in the absence of the 5′‐UTR, whereas mRNA levels were not changed. Moreover, the successive deletion of the first half of the ADAM10 5′‐UTR revealed a striking increase in ADAM10 protein expression, suggesting that this portion of the 5′‐UTR harbours elements that inhibit translation. The study further showed enhanced α‐secretase activity and reduced Aβ expression in conditioned medium expressing both APP and a construct lacking the first half of the ADAM10 5′‐UTR.^86^ Thus, the 5′‐UTR of ADAM10 clearly plays an important role in regulating ADAM10 protein translation.

GC‐rich 5′‐UTRs are characteristic of many genes and are involved in the regulation of gene expression via translational control mechanisms.^87^ Notably, ADAM10 has a GC‐rich 5′‐UTRs, and growing evidence indicates that canonical G‐rich sequence repeat in DNA or RNA tends to form stable G‐quadruplex secondary structures.^88^ By means of circular dichroism spectroscopy, a highly stable and parallel G‐quadruplex secondary structure was observed in the 5′‐UTR of ADAM10. This structure was generated by a G‐rich sequence between 66 nt and 94 nt in the 5′‐UTR.^89^ Mutations of the G‐quadruplex motif resulted in significant increases in ADAM10 protein levels but only small increases in mRNA levels. This finding is similar to those mentioned above regarding the expression of an ADAM10 construct lacking the first half of the 5′‐UTR.^86^

The G‐quadruplex motif is considered a critical RNA secondary structure responsible for the translational repression of ADAM10; thus, genetic variations within or around this motif likely affect ADAM10 translation. Notably, rs653765 is located near the G‐rich sequence in the first half of the ADAM10 5′‐UTR, and variation in this site has been associated with expression changes in soluble APPα, a downstream molecule of ADAM10 signalling.^57^ In addition, according to the Single Nucleotide Polymorphism database (dbSNP), six polymorphic sites are present within the G‐rich sequence in the human genome, namely, rs886775779, rs1022312149, rs367745138, rs900600176, rs373778366 and rs199972921 (Figure 1). However, it is undetermined whether these variations affect ADAM10 translation or confer genetic susceptibility to ADAM10‐related diseases, especially epilepsy.

In addition to transcriptional and posttranscriptional modulation, ADAM10 can be regulated through other mechanisms. For example, TspanC8 tetraspanins are essential regulators of ADAM10 maturation and trafficking to the cell surface.^90^ Based on X‐ray crystallography data, the ADAM10 C‐terminal cysteine‐rich domain partially blocks the enzyme's active site and prevents unfettered substrate access, and the binding of an antibody to this inhibitory structure enhances enzymatic activity.^91^ These findings indicate the potential for other mechanism of ADAM10 activity modulation beyond TFs and miRNAs.

## 5 SIGNIFICANCE OF STAGE‐DEPENDENT ADAM10 IN EPILEPSY

### 5.1 Amyloidogenic processes

Amyloidogenic processes gradually develop with age; thus, dementia and complicated seizures often emerge in the elderly, resulting in a long‐term window for early intervention. As a key α‐secretase, ADAM10 can inhibit the amyloidogenic pathogeneses. Through microarray analyses, ADAM10 overexpression was found to affect a series of pathways, primarily those involved in cell communication, nervous system development, neuronal projections and synaptic transmission. However, proinflammatory and proapoptotic proteins were not up‐regulated in ADAM10 transgenic mice,^37^ indicating that reasonable increases in ADAM10 expression may facilitate seizure control without the risk of significant inflammation‐ and apoptosis‐related damage.

Several TFs, such as XBP1, USF1, Sox2 and PPARα, have been demonstrated to be required for ADAM10 transcription. Thus, these TFs are potential molecules for seizure control. In addition, vitamin A improves the AD‐related attenuation of cognitive function in mouse models by activating the downstream retinoic acid receptor, a TF involved in ADAM10 transcription.^92^ Considering that vitamin A is safe and convenient for administration, the effects of this vitamin on complicated seizures merit identification in future experiments. In addition, the genetic variations that potentially confer susceptibility to epilepsy, such as the C‐A haplotype (rs514049‐rs653765) around the core promoter region 57, 58 and the six polymorphisms within the ADAM10 5′‐UTR G‐rich region must also be studied.

Recently, widespread concern arose over the failure of solanezumab to improve cognitive decline in an AD clinical trial.^34^ Compared to the concentrations in the peripheral blood, the solanezumab concentration in the brain was extremely low because of the blood‐brain barrier (BBB). Thus, there may have been insufficient levels to function, which are considered an important reason for the failure of the trial. Interestingly, miRNAs are key modulators at the posttranscriptional level, having the ability to bypass the BBB via intranasal administration.^93^ Thus, miRNAs might be promising candidates for seizure control. Several miRNAs, such as miR‐144, miR‐221, miR‐1306, miR‐103 and miR‐107, have been observed to function in AD. In addition, as primary drugs for dementia that induce acetylcholinesterase (AChE) overproduction, AChE inhibitors were observed to increase ADAM10 activity by promoting ADAM10 trafficking.^94^ However, it is uncertain whether these molecules are also involved in complicated seizures.

Notably, Adam10dn mice without transgenic APP overexpression experienced seizures for shorter durations and showed less neuronal cell death and neuroinflammation after kainate injection than did wild‐type mice. This result indicates that the antiepileptic effects of ADAM10 that act via amyloidogenic processes most likely depend on APP overexpression.^4^ Hence, the incremental modulation of ADAM10 in amyloidogenic processes seems to be limited to treating epileptic seizures in AD.

### 5.2 Cortical dysplasia

Unlike amyloidogenic processes, cortical development begins during the embryonic period and continues after birth; thus, cortical dysplasia usually occurs because of aetiological injuries during the cortical development. In clinical practice, epileptic seizures triggered by cortical dysplasia are resistant to almost all current drugs. From this perspective, epilepsy should be considered a neurodevelopmental disorder, especially early‐onset epilepsy.^95^ As pathological changes in the cortex are difficult to reverse, surgical resection is considered a strategy to treat cortical dysplasia‐related epilepsy. A series of clinical studies have demonstrated favourable outcome after surgical resection with the help of image positioning.^96^ However, the manifestation of cortical dysplasia is not always typical and is often uncertain in clinical practice. Thus, there is an urgent need to determine how these patients can be identified, particularly in the early processes of cortical dysplasia, as this recognition is necessary for early intervention.

Genetic screening has been widely used for the prenatal diagnosis of developmental disorders in clinical practice, and this approach should supply clues for the early recognition of cortical dysplasia. As ADAM10 is a key modulator of cortical dysplasia, screening for ADAM10 genetic variations might contribute to early recognition of high‐risk individuals with cortical dysplasia‐related epilepsy. In fact, there are numerous polymorphic sites with potentially relevant functionality, such as rs514049, which is near the core promoter region, as well as rs653765, rs886775779, rs1022312149, rs367745138, rs900600176, rs373778366 and rs199972921, all within the ADAM10 5′‐UTR. The association between these polymorphisms and cortical dysplasia warrants future investigation.

In addition to regulatory region polymorphisms, exon mutations are also important; however, missense mutations of ADAM10 in epilepsy patients have not been reported in whole‐exome sequencing clinical studies. These results seem to cast doubt on the genetic involvement of ADAM10 in cortical dysplasia. In our opinion, the negative results of whole‐exome sequencing can be readily explained. Given that ADAM10 is a key modulator of embryonic development, missense mutations in ADAM10 exons most likely result in the severe impairment of its function, followed by embryonic or neonatal death before being enrolled in whole‐exome sequencing.

As mentioned above, surgical resection is an effective therapy for cortical dysplasia, but the development of drugs to block pathologic discharges that result from cortical dysplasia should not be ignored. In recent years, a number of differentially expressed proteins underlying childhood cortical dysplasia with epilepsy were identified via iTRAQ‐facilitated proteomic profiling, which provides a proteomic profile after disease onset.^97^ In addition, miR‐323a‐5p and miR‐4521 expression levels are aberrant in epilepsy patients with focal cortical dysplasia.^98^, ^99^ Hence, in combination with iTRAQ proteomic profiling and genetic screening for the early detection of cortical dysplasia, a comprehensive investigation of these miRNAs might represent a method for treating seizures in these patients.

## 6 CONCLUSIONS

ADAM10 is a key α‐secretase responsible for ectodomain shedding of its substrates. Thus, the function of ADAM10 depends on the biological effects of its substrates. In light of the effects of these substrates, the potential pathogenic mechanisms of ADAM10 in epilepsy are grouped into amyloidogenic processes in the ageing stage and cortical dysplasia in the developmental stage. Therefore, ADAM10 is considered a stage‐dependent modulator, and several considerations for seizure control that make use of multiple modalities were suggested, all of which must be further assessed.

## CONFLICT OF INTEREST

The authors confirm that there is no conflict of interest.
